# Formulation of W/O/W Emulsion-Based Chitosan-Alginate Microcapsules for Encapsulation of Cannabidiol and *A. annua* L. Extract Containing Luteolin and Apigenin: A Response Surface Optimization Approach

**DOI:** 10.3390/pharmaceutics17030309

**Published:** 2025-02-28

**Authors:** Emilija Nemickaite, Ugne Zlabiene, Agne Mazurkeviciute, Mindaugas Marksa, Jurga Bernatoniene

**Affiliations:** 1 Department of Drug Technology and Social Pharmacy, Faculty of Pharmacy, Medical Academy, Lithuanian University of Health Sciences, Sukileliu pr. 13, LT-50161 Kaunas, Lithuania; 2 Institute of Pharmaceutical Technologies, Faculty of Pharmacy, Medical Academy, Lithuanian University of Health Sciences, Sukileliu pr. 13, LT-50161 Kaunas, Lithuania; ugne.cizauskaite@lsmu.lt (U.Z.); agne.mazurkeviciute@lsmu.lt (A.M.); 3 Department of Clinical Pharmacy, Faculty of Pharmacy, Medical Academy, Lithuanian University of Health Sciences, Sukileliu pr. 13, LT-50161 Kaunas, Lithuania; 4 Department of Analytical and Toxicological Chemistry, Medical Academy, Lithuanian University of Health Sciences, LT-50161 Kaunas, Lithuania; mindaugas.marksa@lsmu.lt

**Keywords:** sodium alginate, chitosan, double emulsions, microcapsules, *Artemisia annua* L.

## Abstract

**Background/Objectives:** Chitosan–alginate microcapsules were produced to encapsulate bioactive compounds from *Artemisia annua* L. extract (apigenin, luteolin) and cannabidiol (CBD). The study aimed to optimize emulsion composition and encapsulation parameters for potential applications in food supplements and pharmaceuticals. **Methods:** A water-in-oil-in-water (W/O/W) emulsion and a modified coacervation extrusion technique were employed. The study was conducted in two phases using response surface methodology. Key metrics included encapsulation efficiency (EE), yield (EY), cumulative release in vitro, and physicochemical and morphological properties, analyzed via scanning electron microscopy (SEM), Fourier transform infrared spectroscopy (FT-IR), high-performance liquid chromatography with a diode array detector (HPLC-DAD), and gas chromatography with flame ionization detection (GC-FID). **Results:** The optimal conditions were identified as 0.1% Tween 20, 3.8% Span 80, 3.8% CBD, 19.9% *A. annua* L. extract, 1.5% outer-phase Tween 20, 48.5% sodium alginate, 200 rpm stirring for 30 min, and a 0.05 mL/min flow rate. The EE values were 80.32 ± 4.11% for CBD, 88.13 ± 3.13% for apigenin, and 88.41 ± 4.17% for luteolin, with respective cumulative releases of 77.18 ± 4.4%, 75.12 ± 4.81%, and 75.32 ± 4.53%. **Conclusions:** The developed microcapsules demonstrated high encapsulation efficiency and controlled release, highlighting their potential for further development in food supplements and pharmaceuticals. Future studies should focus on refining the formulation for improved bioavailability and stability.

## 1. Introduction

In recent years, phytotherapy—defined as the therapeutic use of whole or minimally modified plant components—has ignited significant scientific interest. *Artemisia annua* L., a member of the *Asteraceae* family, possesses an abundant profile of polyphenols, coumarins, sesquiterpenes, and flavonoids, including the flavones apigenin and luteolin [1,2,3]. It has already been proven that extracts of *Artemisia annua* L. exhibit strong anti-inflammatory effects. Furthermore, it is either currently used or under investigation for its potential in treating cancer and malaria [4,5,6,7].

Similarly, *Cannabis sativa* L. is known for its diverse composition, containing terpenes, alkaloids, phenolic compounds, and cannabinoids, with notable components such as cannabinol (CBN) and cannabidiol (CBD) [8]. Over the past decade, the medicinal properties of *Cannabis sativa* L. have been widely studied. It is known for its ability to induce relaxation and is applied in the treatment of serious illnesses, including glaucoma, depression, neuralgia, multiple sclerosis, Alzheimer’s disease, and the alleviation of symptoms related to HIV/AIDS and cancer [8,9]. CBD alone exhibits diverse therapeutic effects, such as neuroprotection, antiepileptic activity, anxiolytic and antipsychotic properties, as well as anti-inflammatory, analgesic, and anticancer properties [10].

Combining the chemical constituents of *Cannabis sativa* L. and *Artemisia annua* L. could present innovative opportunities for disease prevention and treatment research. To the best of our knowledge, no previous studies have investigated the synergistic potential of these two plant species.

Microencapsulation, a process that employs polymers to create a physical barrier around active components, is a widely used technique to enhance stability, reduce volatility, and control the release of bioactive compounds [11,12,13,14]. Various encapsulation methods are available, including physicochemical methods such as simple or complex coacervation, physical methods like spray-drying, and chemical methods such as interfacial polymerization [11,12,13,14,15].

Complex coacervation is a promising microencapsulation technique that has been extensively employed in various industries, including pharmaceuticals, food, agriculture, and textiles [16]. Studies have shown that the combination of sodium alginate and chitosan results in alginate–chitosan-coated microcapsules with complementary physical properties, leading to stronger, more elastic, and versatile capsules [17,18].

In microencapsulation via complex coacervation, emulsions often serve as the basis for incorporating active compounds. Multiple emulsions are particularly advantageous for encapsulating active compounds within inner hydrophilic and lipophilic phases. Research has suggested that the co-encapsulation of multiple bioactive components significantly enhances their bioactivity and functionality compared to single components [19]. However, the use of sodium alginate and chitosan in multiple water-in-oil-in-water (W/O/W) emulsions has been less studied than oil-in-water or water-in-oil emulsions, likely due to the thermodynamic instability of bilayer emulsions and osmotic pressure gradients that can result in swelling and rupture [20]. Therefore, the careful optimization of parameters such as encapsulation efficiency, release profile, and the physical and morphological properties of microcapsules is critical. Notably, Yi Li et al. proposed that W/O/W emulsion encapsulation using chitosan–alginate beads is a promising approach for precisely delivering bioactive ingredients to the intestines [21].

In this study, we aim to investigate and optimize the formation of alginate–chitosan-coated microcapsules using a complex coacervation extrusion technique with W/O/W emulsions. These microcapsules will encapsulate the flavones apigenin and luteolin alongside the cannabinoid CBD as the primary active compounds. Our focus is to optimize both the emulsion composition and the microencapsulation process parameters through a surface response approach. The aim is to consider various morphological, physical, and pharmaceutical factors to develop a sub-product suitable for pharmaceutical applications.

## 2. Materials and Methods

### 2.1. Materials

The dried *Artemisia annua* L. herbs were purchased from UAB “Žolynų Oazė” (Vilkaviškio raj, Lithuania). Cannabis seed oil was purchased from UAB “Strazdų žaliasis auksas” (Širvintų raj, Lithuania). The chemicals used for this research were acetic acid 99.8–110.5% purchased from Honeywell Fluka (Seelze, Germany); calcium chloride and medium-molecular-weight (MMW) chitosan (degree of deacetylation (DDA): approximately 75–85%; molecular weight: 190,000–310,000 Da) purchased from Sigma-Aldrich (Darmstadt, Germany); crystal-resistant distillate cannabidiol (CBD) purchased from Fitodenta (Kaunas, Lithuania); 96% ethanol (Vilnius, Lithuania), L-glutathione ≥ 98% (ROTH, Karlsruhe, Germany), phosphate-buffered saline (PBS) solution, and sodium alginate (molecular weight: 80,000–120,000 Da) purchased from TCI (Zwijndrecht, Belgium); Span 80 and Tween 20 purchased from Sigma-Aldrich (Steinheim am Albuch, Germany).

### 2.2. Preparation of Artemisia annua L. Extract

The dried herb of the *Artemisia annua* L. was milled using a trapezoid 0.5 mm hole sieve at 800 rpm using the Ultra Centrifugal Mill ZM 200 (Retsch, Haan, Germany). The ground plant material was soaked in 80% ethanol (*v*/*v*), and 0.25 mg L-glutathione was used as the excipient for the extraction process. The sample was later sonicated in the ultrasound water bath “Grant XUB10” (Grant Instruments, Cambridge, UK) and centrifuged at 3000 rpm for 15 min using “Sigma 3-18KS” (SIGMA Laborzentrifugen, Osterode am Harz, Germany). Sonication parameters (200W, 38 kHz, 25 °C, for 30 min) were chosen to ensure effective extraction of bioactive compounds while avoiding thermal degradation. These conditions are consistent with previous findings that suggest 25 °C minimizes polyphenol oxidation during extraction. Finally, the sample was filtered with filter paper (MN 617, Macherey-Nagel, Düren, Germany) to obtain a clear extract [22].

### 2.3. W/O/W Emulsion Composition

When preparing the primary emulsion (W/O), the water phase consisted of the *Artemisia annua* L. extract, Tween 20, and 1% chitosan. The oil phase was composed of cannabis seed oil, Span 80, and crystal-resistant CBD distillate. The primary emulsion was homogenized by stirring at 1800 rpm using an IKA Eurostar 200 (IKA Works, Staufen, Germany) and then sonicated at 200 W for 10 min, maintaining a temperature of up to 20 °C. The water and oil phase ratio was 2:3 accordingly.

The outer water phase was prepared using 1% sodium alginate and Tween 20. The W/O emulsion was subsequently mixed with the sodium alginate solution and stirred at 800 rpm while being sonicated for 5 min at 20 °C. The ratio of W/O emulsion to outer water phase was kept constant at 1:1.

Solutions of 1% sodium alginate and medium-molecular-weight (MMW) chitosan were prepared separately using a magnetic stirrer (Windaus Labortechnik GmbH, LED 2002, Clausthal-Zellerfeld, Germany) at 300 rpm for 30 min and then allowed to swell overnight.

### 2.4. Preparation of Microcapsules

A 2% calcium chloride (CaCl_2_) solution was mixed with the chitosan solution in a 1:1 ratio. The chitosan solution was neutralized with a 10% sodium hydroxide (NaOH) solution to a pH of 6.5 to activate the gelation properties of the chitosan. The double W/O/W emulsion containing sodium alginate was then loaded into a 10 mL syringe and mounted on a syringe pump (Landgraf Laborsysteme HHL, LA-120, Langenhagen, Germany). The emulsion droplets were introduced into the crosslinking CaCl_2_ solution containing the neutralized chitosan while being stirred with a magnetic stirrer (Windaus Labortechnik GmbH, LED 2002, Clausthal-Zellerfeld, Germany). The microencapsulation conditions varied throughout the study. After the microencapsulation process, the microcapsules were filtered, washed with purified water, and left to dry at room temperature for 48 h.

### 2.5. Surface Response Design

To optimize the conditions for emulsion composition and the preparation of chitosan–alginate microcapsules, Design Expert software (v. 13.0.5.0, Stat-Ease, Inc., Minneapolis, MN, USA) was utilized. The selected approach was a surface response (optimal) design to establish the relationship between variables and responses.

For the formulation of the double W/O/W emulsion, five variables were selected (Table 1). The responses evaluated included the creaming index, droplet size in the inner and outer phases, viscosity, encapsulation yield, and the encapsulation efficiency for CBD, apigenin, and luteolin. The concentrations of sodium alginate and chitosan solutions were kept constant. A total of 31 experiments were generated based on the design.

For optimizing the chitosan–alginate microcapsule preparation technique, the variables used are listed in Table 2. The responses evaluated included the microcapsule size, moisture content, bulk density, compression properties, encapsulation yield, encapsulation efficiency for CBD, apigenin, and luteolin, as well as the release profiles of these compounds. A total of 25 experiments were designed and conducted.

### 2.6. Creaming Index (CI) Determination

The creaming index analysis of the double emulsion was performed using a centrifuge (Sigma 3-18KS, SIGMA Laborzentrifugen, Osterode am Harz, Germany). Two grams of each sample were placed into 2 mL centrifuge tubes, and their initial mass was recorded (m_0_). The samples were centrifuged at 3000 rpm for 5 min. Following centrifugation, the oil layer at the surface of each tube was removed, and the tubes were weighed again (m). The analysis was repeated three times, and the average value was calculated [23]. The creaming index was determined using the following equation:

CI (%) = ((m_0_ − m)/m_0_) × 100 (1)

### 2.7. Viscosity Determination

The viscosity of the double emulsion was measured by the rotational viscosimeter Alfa L series (Fungilab, Barcelona, Spain) equipped with L4 spindle (length 115 mm, diameter 4 mm). The sample was poured into a tall thin tube and placed under the viscosimeter spindle, and the viscosity was measured at 100 rpm rotational speed and 22 s^−1^ shear rate. Measurements were carried out at room temperature after 15 s. Results are provided in mPa∙s [24].

### 2.8. Inner and Outer Emulsion Droplet and Microcapsule Size Determination

The inner droplet size of the emulsion was analyzed and measured using a Motic^®^ BA310 microscope (Nis-Elements, BMS, Barcelona, Spain) equipped with Nis-Elements imaging software (Version 7.1.0.3, BMS, USA) for image evaluation. The size of the outer droplets was determined using a Mastersizer 3000 (Malvern Panalytical Ltd., Malvern, UK). Additionally, images of the emulsion were captured with a Nikon Eclipse 50i microscope (Nikon Co., Tokyo, Japan) at 10× magnification.

The outer droplet size was measured using the Mastersizer 3000 with a Hydro EV unit (Malvern Panalytical Ltd., Malvern, UK). Samples were added dropwise into water to achieve laser obscuration levels between 10.5% and 11.5%. A refractive index of 1.452 was used for the dispersing material, with a density of 0.922 g/cm^3^. The pump speed was maintained at a constant 2400 rpm. Particle size distribution was assessed in five runs, and the average was calculated. The formulations were characterized by the percentile D50 values [25,26].

Microcapsule size was determined using the Motic^®^ BA310 microscope at 10× magnification. Five randomly selected microcapsules per sample were measured, and the mean size was calculated. All measurements are reported in micrometers (µm) and millimeters (mm).

### 2.9. HPLC Analysis of Apigenin and Luteolin

The predominant active compounds were identified using high-performance liquid chromatography (HPLC). The analysis was performed on a Waters 2695 chromatographic system equipped with a Waters 996 diode array detector (Milford, MA, USA) and an ACE 5C18 chromatography column (250 × 4.6 mm). Data were processed using Empower 3, Waters Chromatography Data Software Version 3.8.0. The eluent system consisted of 0.1% trifluoroacetic acid as eluent A and 100% acetonitrile as eluent B. The elution program was as follows: 5% to 15% B from 0 to 8 min, 15% to 20% B from 8 to 30 min, 20% to 40% B from 30 to 48 min, 40% to 50% B from 48 to 58 min, 50% to 50% B from 58 to 65 min, 50% to 95% B from 65 to 66 min, 95% to 95% B from 66 to 70 min, and 95% to 5% B from 70 to 71 min. The injection volume was 10 µL, the column temperature was maintained at 25 °C, and the mobile phase flow rate was 1 mL/min, with a total run time of 81 min. The absorption of apigenin was measured at 340 nm, while luteolin was measured at 350 nm. Quantification was performed using the external standard method. Calibration curves were constructed, with R^2^ values of 0.999383 for luteolin and 0.998872 for apigenin.

### 2.10. GC Analysis of CBD

Gas chromatography with a flame ionization detector (FID) analysis was conducted on the Shimadzu GC-2010 Plus (Tokyo, Japan). An Rxi-5 MS capillary column (30 m length, 0.25 mm inner diameter, and film thickness of 0.25 μm) was utilized. The temperature was programmed starting at 80 °C for 1 min, then ramped up to 250 °C at a rate of 10 °C/min. It was further increased to 310 °C at a rate of 30 °C/min and held for 7 min. The total run time was 30 min. Helium was used as the carrier gas at a flow rate of 1 mL/min. The detector temperature was maintained at 330 °C. The injection mode was split (1:10), with an injection volume of 1 µL.

### 2.11. EE and EY of Active Ingredients Determination

Encapsulation yield (EY%) is calculated by dividing the weight of the obtained microcapsules by the weight of the multiple emulsion used to form the microcapsules, accounting for any residual material remaining in the syringe. EY is expressed as a percentage and calculated using the following formula:

EY (%) = (m_0_/(m_1_ − m_2_)) × 100, (2)

where:

m_0_—weight of freshly made microcapsules;

m_1_—weight of an empty syringe;

m_2_—weight of the syringe filled with the emulsion.

The encapsulation efficiency (EE%) was determined based on the methodology provided by Aitor Villate and Nasim Khorshidian [27,28]. Specifically, 200 mg of microcapsules were dissolved in 2 mL phosphate buffer solution (PBS). The samples were sonicated for 1 h at 30 °C. The sample was then extracted with 20 mL of ethanol and centrifuged at 3000 rpm for 5 min. Finally, the supernatant was filtered into HPLC analytical flasks using a 0.45 µm membrane filter, and high-performance liquid chromatography (HPLC) was conducted.

The encapsulation efficiency (EE%) of apigenin, luteolin, and CBD was calculated using the following equation:

EE (%) = (C_0_/C_i_) × 2 × 20 × 100 (3)

where:

C_0_—determined compound concentration;

C_i_—the initial compound concentration.

### 2.12. Moisture Content Determination

A Moisture Analyzer DAB (KERN & SOHN Gmb, Balingen, Germany) was used to determine the moisture content of the microcapsules. Microcapsules (0.2 ± 0.05 g) were dried at 105 °C until complete moisture evaporation and until the weight remained constant. The test was repeated three times, and the average of the results, expressed as a percentage (%), was calculated [25].

### 2.13. Compression Test

The compression test was conducted with the texture analyzer TA.XT Plus (Stable Micro Systems, Godalming, UK). Five freshly made microcapsules of each sample were placed on plate and used for the analysis. The flat base attached to the device descended, pressed the microcapsules, then returned to its starting position. The test was repeated five times for each series and the program automatically calculated the average and created a graph of the results.

The parameters for the analysis were pre-test speed 1 mm/s, test speed 2 mm/s, post-test speed 10 mm/s, distance 1 mm, and trigger force 5 g. The maximum force of the device was set to 6500 g. The compression was measured in g of hardness [29].

### 2.14. Bulk Density Determination

The bulk density of the microcapsules was assessed using a conical volumeter (Copley Scientific, BEP2, Nottingham, UK). Five grams of microcapsules were placed into a metallic funnel and subjected to 20 s of vibration. Afterward, the lower lid of the funnel was opened, and the time taken for all the microcapsules to pass through was recorded (t). The bulk density was expressed in g/s.

### 2.15. Release In Vitro of Active Ingredients Determination

The dissolution and the release of the microcapsule compounds (apigenin, luteolin, and CBD) were determined according to the European Pharmacopoeia article (Eur. Ph 2.9.3.). Two grams of microcapsules were placed in the basket stirring element and were later immersed in the SOTAX CH-4147 (Aesch, Switzerland) apparatus cylinder with the simulated gastric medium (pH 1.5). The samples were collected in 30 min intervals (30 min, 60 min, and 90 min) and filtered through the 0.45 μm membrane filter. Later, the medium was changed to the simulated intestine medium (pH 7.5). The samples were again collected in 30 min intervals (30 min, 60 min, 90 min, and 120 min) and filtered through the 0.45 μm membrane filter. Over the course of 6 h, the microcapsules were stirred at 50 rpm speed at 37 ± 0.2 °C. The stimulated gastric medium and the stimulated intestinal medium were prepared according to the European Pharmacopoeia article (Eur. Ph 5.17.1). The samples were analyzed with high-performance liquid chromatography (HPLC).

The in vitro release data were applied to the following kinetic models: zero order, first order, Higuchi, and Korsmeyer–Peppas to predict the mechanism and kinetics of CBD, luteolin, and apigenin from microcapsules. The model that gave the highest correlation coefficient value was considered as the best fit of the release data [30].

### 2.16. SEM Analysis of Microcapsules

The surface morphology and structure of the chitosan-coated microcapsules were examined using a scanning electron microscope (SEM) (S-3400N, Hitachi Science & Technology, Tokyo, Japan) at an accelerating voltage of 5.0 kV. The microcapsules were mounted on double-sided adhesive tape on a sample holder. The chamber was evacuated, and images were captured at magnifications ranging from 25× to 1000× at room temperature.

### 2.17. FT-IR Analysis of Microcapsules

The FT-IR analysis of the microcapsules was conducted using an FT-IR spectrophotometer (Tensor 27, Bruker, Billerica, MA, USA). The samples were scanned 31 times over a wavelength range of 4000 to 400 cm⁻^1^ with a resolution of 4 cm⁻^1^. The peaks were measured in absorbance units for quality analysis.

### 2.18. Statistical Analysis

The data were presented as mean ± standard deviation (SD). The statistical analysis was performed using SPSS (V. 29.0.0.0, IBM, Chicago, IL, USA), and the response surface test data were statistically analyzed using Design Expert program (V. 13.0.5.0, Stat-Ease, Inc., Minneapolis, MN, USA). The level of significance was valued at *p* < 0.05.

## 3. Results and Discussion 

To identify the optimal technological conditions and W/O/W emulsion composition for chitosan–alginate microcapsules, a response surface design approach was employed. The results are as follows.

### 3.1. Surface Response Design Approach for Optimal W/O/W Emulsion Composition

To optimize the emulsion composition for achieving the highest yield and encapsulation efficiency of the bioactive compounds from *A. annua* L. (luteolin and apigenin) and *C. sativa* (CBD) in microcapsules, a response surface experimental matrix comprising 31 emulsion compositions was generated using Design Expert software. At this stage, the concentration of sodium alginate was excluded from consideration.

The matrix, model fit, and significance were thoroughly analyzed. A polynomial model with a linear process order was selected. For a valid lack-of-fit test, it is recommended to have at least three degrees of freedom for lack of fit and four for pure error; in this study, these values were 20 and 5, respectively. The alias equations for the different terms are presented in Table 3.

#### 3.1.1. Creaming Index (CI) Determination

First, the stability of the emulsions was assessed using the calculated creaming index (%). Out of the 31 samples, 12 exhibited instability, with creaming index values ranging from 2.0 ± 0.1% to 17.1 ± 0.9%. A square root transformation of the data was applied and analyzed using the suggested ANOVA quadratic model. However, the model’s level of significance was 0.0349, and the lack-of-fit *p*-value was 0.0556, indicating that the model did not fit well due to noise levels (ranging from 5.56% to 7.76%).

**Figure 1:**
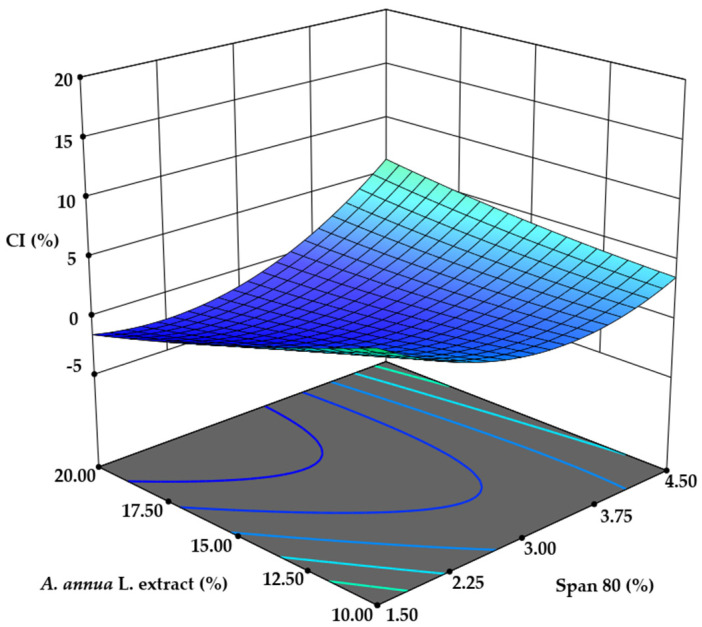
The impact of *A. annua* L. extract and Span 80 concentration on the CI of emulsions.

Despite this, one term, BD, was considered significant based on its *p*-value (Figure 1). This result is not unexpected, as emulsion stability depends on the type and concentration of emulsifiers, as well as the hydrophilic-to-lipophilic phase ratio used during emulsion formation [23,31].

#### 3.1.2. W/O/W Emulsion Viscosity Determination

Further analysis was conducted on the viscosity of the prepared samples. The emulsions exhibited a viscosity range from 450.8 mPa∙s to 970.4 mPa∙s at room temperature and with a shear rate of 22 s^−1^. The volume fraction of the inner aqueous phase in the final W/O/W emulsion was 20%, calculated based on the ratio of the dispersed inner phase (*A. annua* L. extract + Tween 20 + chitosan) to the total emulsion volume. Linear ANOVA analysis revealed a *p*-value of 0.0482, indicating statistical significance. Spearman’s correlation coefficient (r = 0.420) showed a statistically significant relationship between *A. annua* L. extract concentration and emulsion viscosity. This term was also identified as the only significant factor in the surface response model.

Interestingly, variations in CBD concentration did not significantly affect emulsion rheology, contrary to findings by S. Martinez et al. (2022), which indicated that higher oil phase concentration and density typically increase emulsion viscosity [32]. However, in this study, reducing CBD content—a highly viscous ingredient—by 50% resulted in a viscosity reduction from 960.5 mPa∙s to 727.8 mPa∙s in certain samples (*p* < 0.05).

#### 3.1.3. W/O/W Emulsion Inner and Outer Droplet Size Determination

Continuing the research, the morphology of the primary W/O emulsion and the final W/O/W emulsion used for microcapsule extrusion was evaluated. It is well-established by Q. Pen et al. that both the release characteristics and the stability of encapsulated active ingredients are strongly influenced by the emulsion droplet size and its distribution. Smaller and more uniform droplet sizes result in more stable microcapsules [33].

The emulsion droplet size of the inner W/O emulsion was first evaluated through microscopic analysis, revealing a size range of 0.302 μm to 0.408 μm, depending on the emulsion composition. When the concentrations of CBD and *A. annua* L. extract were doubled, the droplet size increased significantly from 0.345 ± 0.016 μm to 0.375 ± 0.019 μm (*p* < 0.05). Studies have shown that increasing ethanol concentrations in emulsion formulations can lead to larger droplet sizes and higher ζ-potential values [34,35]. A similar trend was observed in this study: reducing CBD content by 50%, a highly viscous component, led to a 9.86% decrease in emulsion droplet size, likely due to the density of CBD.

**Figure 2:**
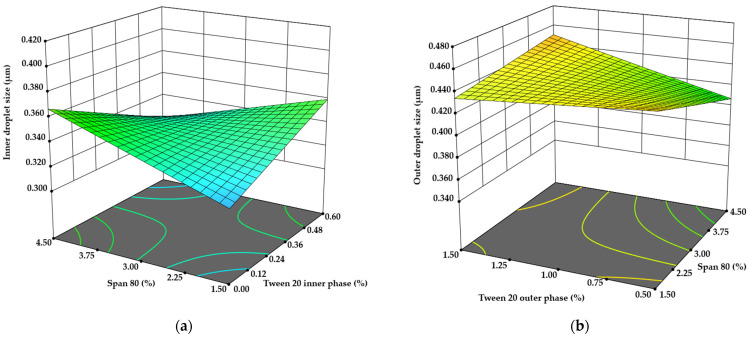
The influence of emulsifiers on (**a**) W/O and (**b**) W/O/W droplet size.

According to B.P. Binks et al., differences in droplet size can lead to creaming or sedimentation of the emulsion, depending on the density differences between the dispersed and continuous phases. This can be enhanced or restricted by flocculation [36]. In this study, inner droplet size data analysis using the sequential sum of squares for the two-factor interaction model (2FI) showed that active ingredients alone did not directly correlate with droplet size (*p* = 0.0215). However, the emulsifiers, Span 80 (*p* = 0.0263) and Tween 20 (*p* = 0.0096), as well as some interaction terms (AB, AC, BD), significantly influenced droplet size (*p* = 0.0012, *p* = 0.0068, and *p* = 0.0061, respectively; Figure 2a). This result aligns with the established understanding that emulsifier concentrations and the hydrophilic–lipophilic balance significantly affect emulsion stability [37].

Analysis using the Mastersizer 3000 (Malvern Panalytical Ltd., Malvern, UK),indicated that the majority of the outer droplet sizes in the W/O/W emulsion fell within the range of 0.3–1.0 μm, with an actual range of 0.350 μm to 0.471 μm, qualifying the emulsion as a nanoemulsion. Applying the same 2FI ANOVA analysis to the outer droplet size dataset revealed a significant model (*p* = 0.0396) with a lack-of-fit value of 0.3052. Significant terms included D, AC, and BE (*p* = 0.0278, *p* = 0.0155, and *p* = 0.0157, respectively). While the emulsifiers did not exhibit a linear correlation with droplet size, the stabilization of the emulsion with sodium alginate at this step made the interaction between Span 80 and Tween 20 more significant (Figure 2b).

Interestingly, reducing the ethanolic extract of *A. annua* L. by 50% resulted in a 5.18% increase in the outer droplet size (*p* < 0.05). These findings contradict previous studies that observed reductions in droplet size with similar formulations [34,35]. This discrepancy may be attributed to the saponin content in *A. annua* L. extract. Saponins have recently garnered attention for their potential as natural emulsifiers [38]. According to M. Jarzebski et al., the addition of saponins up to 2 g/L in emulsion systems significantly decreases both the creaming index and particle size distribution [39].

#### 3.1.4. Encapsulation Efficiency (EE) and Encapsulation Yield (EY) Evaluation

Further, the critical characteristics of encapsulation efficiency and encapsulation yield of bioactive ingredients in microcapsules prepared from W/O/W emulsions were evaluated. The surface response design matrix analysis applied a linear model for EY (*p* = 0.0169). Significant terms influencing EY included the concentrations of Span 80 and Tween 20 in the outer aqueous phase. Pearson’s correlation coefficients indicated a strong correlation between Span 80 concentration and EY (r = 0.520), while Tween 20 in the outer aqueous phase exhibited a moderate correlation (r = 0.341). The EY values ranged from 86.21 ± 4.55% to 98.29 ± 4.70% throughout the experiments. Increasing Span 80 concentration three-fold resulted in a 3.84% higher EY, whereas the same increase in Tween 20 concentration led to only a 2.51% increase.

Encapsulation efficiency of the active ingredients was also examined, as EE is a key parameter in microencapsulation. Ideally, full encapsulation of the extract with the wall material would allow the active compound content in microcapsules to be calculated directly from the emulsion composition. However, in practice, not all materials are fully encapsulated. The actual amounts of luteolin, apigenin, and CBD in the microparticles were quantified using the HPLC and gas chromatography methods described above (see Section 2.9 and Section 2.10).

**Figure 3:**
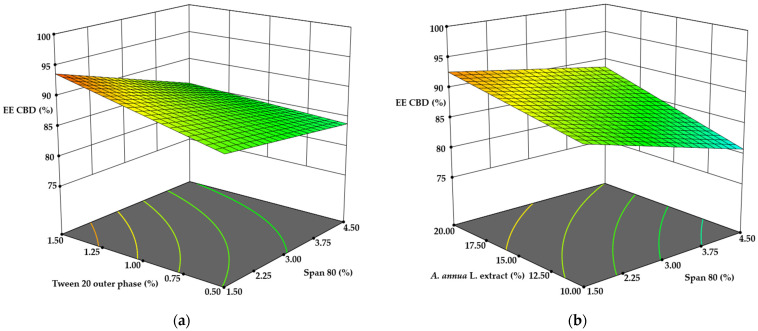
The influence of (**a**) emulsifiers and (**b**) *A. annua* L. extract on the encapsulation efficiency of CBD.

The EE of CBD ranged from 80.98 ± 2.0% to 93.13 ± 3.7%. Surprisingly, the inclusion of CBD in the emulsion did not significantly influence its encapsulation efficiency, while emulsifier concentrations had a considerable impact. It was expected that increasing CBD content in the emulsion would significantly enhance EE, but although differences were observed, they were not statistically significant, ranging from 88.15 ± 4.4% to 93.67 ± 4.7%. A linear ANOVA analysis of the surface response design (*p* = 0.0041) indicated that CBD encapsulation efficiency was affected by the concentrations of Span 80, *A. annua* L. extract, and Tween 20 in the outer aqueous phase. A 200% increase in Span 80 concentration resulted in a 5.21% decrease in EE, while the same increase in Tween 20 concentration improved EE by 4.33% (*p* < 0.05) (Figure 3a). Further investigation is needed to understand the impact of *A. annua* L. extract on CBD encapsulation. Doubling the concentration of *A. annua* L. extract led to a 4.13% improvement in EE (*p* < 0.05) (Figure 3b). It is hypothesized that this effect may be attributed to the saponins present in the extract. Studies have shown that saponins can enhance the stability of phytocannabinoid nanoemulsions [39,40].

**Figure 4:**
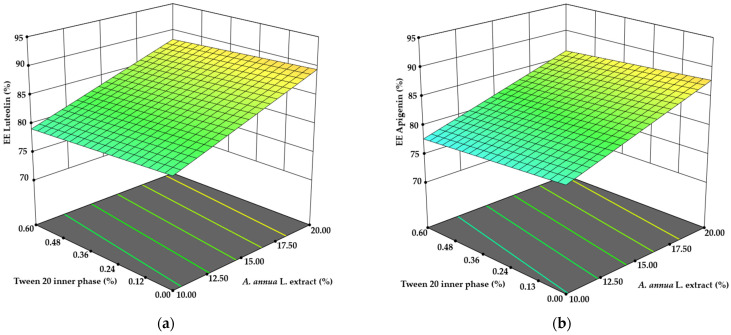
The influence of *A. annua* L. extract concentration on the encapsulation efficiency of (**a**) luteolin and (**b**) apigenin.

The only significant correlation regarding the encapsulation efficiency of luteolin and apigenin was observed with the concentration of *A. annua* L. extract. Pearson’s correlation coefficients were determined as 0.748 for apigenin and 0.670 for luteolin. Both data sets were analyzed using linear ANOVA, yielding *p*-values of 0.0029 for luteolin and 0.0001 for apigenin. In both cases, the concentration of plant extract was identified as the sole significant term (*p* < 0.0001). Increasing the extract concentration in the emulsion composition by 75% resulted in an EE improvement of 5.89% for luteolin and 6.43% for apigenin (*p* < 0.05) (Figure 4).

#### 3.1.5. The W/O/W Emulsion Optimization

**Figure 5:**
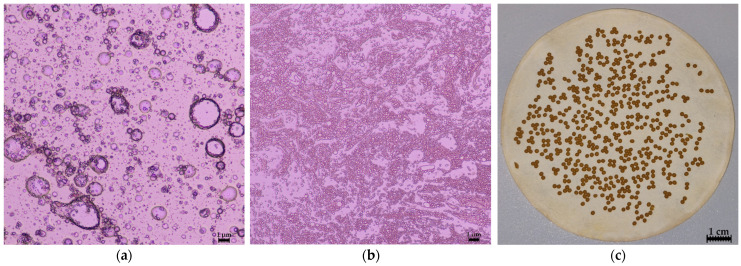
Microscopic images of the (**a**) W/O emulsion and (**b**) W/O/W emulsion under 10× magnification. (**c**) Microcapsules after the composition optimization.

The optimization of the emulsion composition was conducted to identify the most effective formulation. The numeric optimization of the double (W/O/W) emulsion composition was selected, and the importance levels of the output parameters were assigned. The encapsulation efficiency of active ingredients was given the highest priority (5+), followed by encapsulation yield (4+). Viscosity, inner droplet size, and outer droplet size were assigned medium importance (3+), while the creaming index was given low importance (2+). The optimal W/O/W emulsion composition (further referred to as Composition A), achieving a desirability value of 0.753, was determined to include the following parameters: inner aqueous phase Tween 20 concentration at 0.1%, Span 80 at 3.8%, CBD at 3.8%, *A. annua* L. extract at 19.9%, and outer aqueous phase Tween 20 at 1.5%. This optimal composition demonstrated an encapsulation efficiency of CBD (90.03 ± 4.12%), apigenin (87.09 ± 3.13%), and luteolin (89.12 ± 4.25%), along with an encapsulation yield of 93.18 ± 2.60%. The viscosity was measured at 852.7 ± 128.0 mPa·s, inner droplet size at 0.351 ± 0.019 μm, and outer droplet size at 0.418 ± 0.017 μm. The creaming index (CI) could not be accurately predicted due to data transformation. Validation experiments confirmed that the obtained results did not differ significantly from the predicted values, indicating that the optimization process was successful. The microscopic images of the prepared emulsions and microcapsules are shown in Figure 5. Notably, the CI of the optimized sample was 0%.

### 3.2. Surface Response Design Approach for Process Optimization of Alginate–Chitosan Microcapules Prepared Using W/O/W Emulsion

The optimized emulsion composition was further used in the second phase of our study to evaluate the impact of various technological encapsulation parameters, such as stirring speed after extrusion, stirring time, microcapsule extrusion speed, and sodium alginate concentration on the quality of microcapsules. For this purpose, a second surface response D-optimal design matrix was generated, consisting of 25 experiments. The following outcomes were measured: encapsulation yield, encapsulation efficiency, release profile, microcapsule size, firmness, moisture content, and bulk density. The model fit and significance were analyzed. The chosen model type was polynomial, and the process order was linear. The alias equations for different terms are presented in Table 4.

#### 3.2.1. Microcapsule Moisture Content Determination

**Figure 6:**
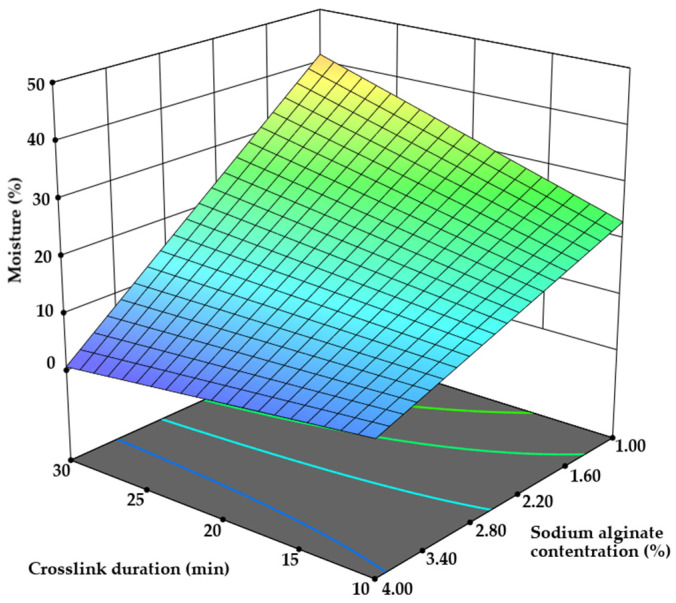
The influence of sodium alginate and crosslink duration on the moisture content of microcapsules.

First, the physicochemical characteristics of the prepared microcapsules were evaluated. Since microcapsules are often used as intermediates in pharmaceutical technology and are later compressed into tablets, their properties such as moisture content, resistance to compression, and bulk are critical factors to consider. The moisture content of the samples varied significantly. Moisture is an essential parameter in evaluating microcapsule quality and shelf life. High moisture levels can lead to stickiness in the particles, causing aggregation, collapse, and even oxidation of encapsulated bioactive ingredients [41]. It was found that samples prepared with 1.00% sodium alginate concentration exhibited moisture contents between 22.03 ± 1.10% and 47.15 ± 2.35%. In contrast, microcapsules with sodium alginate concentrations of 2.00–2.94% demonstrated moisture levels ranging from 15.67 ± 0.75% to 25.06 ± 1.25%, while those with 3.20–3.64% displayed moisture levels between 8.71 ± 0.40% and 15.30 ± 0.75%. The moisture content data were analyzed using the sequential sum of squares for a two-factor interaction ANOVA, yielding a *p*-value of 0.0001. It was revealed that terms B, C, D, and BD had significant influences on moisture content. Microcapsules formulated with a 4% sodium alginate concentration had moisture contents ranging from 3.10 ± 0.15% to 7.31 ± 0.35% (*p* < 0.05 vs. 1–2% sodium alginate) (Figure 6). It is recommended that moisture content should be maintained below 4–5% [42]. The Spearman correlation coefficient between moisture content and sodium alginate concentration was 0.929. Additionally, increasing stirring time from 10 to 30 min led to a 6.07% increase in moisture content, whereas a five-fold increase in extrusion volume resulted in only a 6.35% increase (*p* < 0.05).

#### 3.2.2. Microcapsule Texture Analysis

**Figure 7:**
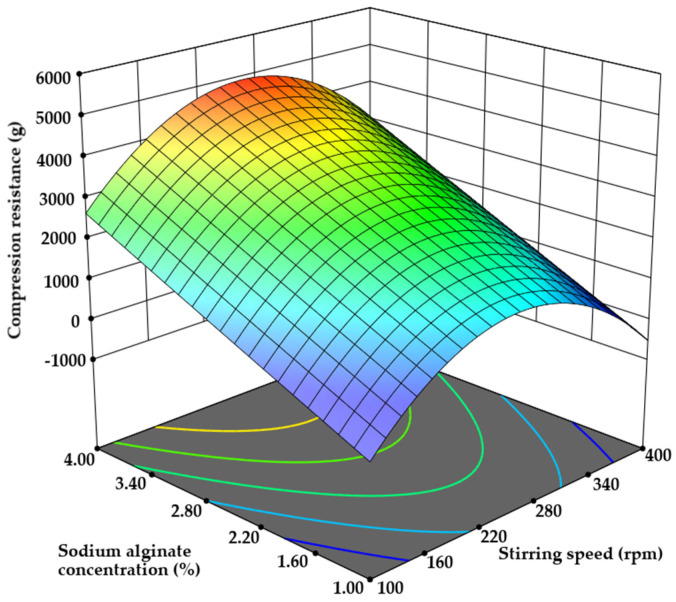
The influence of sodium alginate and stirring speed on the compression resistance of microcapsules.

A thorough examination of the microcapsules’ compression resistance data revealed that microcapsules with 1.00% sodium alginate exhibited significant hardness ranging from 346 ± 17 g to 771 ± 39 g, depending on other technological conditions. In contrast, microcapsules prepared with a 4% sodium alginate solution concentration demonstrated the highest hardness, measuring from 3060 ± 153 g to 5111 ± 256 g (r = 0.849, *p* < 0.05). Both quadratic and linear models were found to be suitable according to the analysis conducted using surface response design software. However, the quadratic model was chosen, with a *p*-value of 0.0028, and terms D, BC, and A^2^ (see Section 2.5 Table 2) were identified as significant model terms. As previously mentioned, a three-fold increase in sodium alginate concentration results in a 135.05% increase in the force required for microcapsule compression. This result aligns with other studies, which have established that the type and concentration of polymers significantly influence the mechanical properties of the polymeric coating of microparticles. High molecular weights or higher concentrations used in the emulsion formation process enhance the mechanical stability of the microcapsules [17,43] (Figure 7).

Stirring conditions also had a significant impact on compression resistance. Increasing stirring speed from 100 to 250 rpm resulted in over 200% higher compression resistance. However, increasing the stirring speed to 400 rpm demonstrated only a 25% increase in compression resistance (*p* < 0.05). Higher stirring speeds improve the even distribution of chitosan and calcium chloride with alginate, enhancing the firmness by reinforcing the alginate network and promoting a more uniform crosslinking process. Conversely, excessive stirring can lead to shear forces breaking the alginate microcapsules, resulting in less mechanically stable structures [44,45].

Furthermore, the interaction between flow rate and stirring duration also influenced microcapsule resistance. When the microcapsule flow rate was reduced from 0.05 to 0.01 mL/min and the stirring time was set to 10 min, the firmness increased by 24.19%. However, extending the stirring duration to 30 min with a lower flow rate (0.01 mL/min) resulted in a 38.52% decrease in firmness (*p* < 0.05 vs. 0.05 mL/min flow rate). This outcome suggests that at lower flow rates and prolonged stirring times, less controlled gelation occurs, leading to areas of weaker crosslinking and reduced firmness [46,47].

#### 3.2.3. Microcapsule Bulk Density Determination

**Figure 8:**
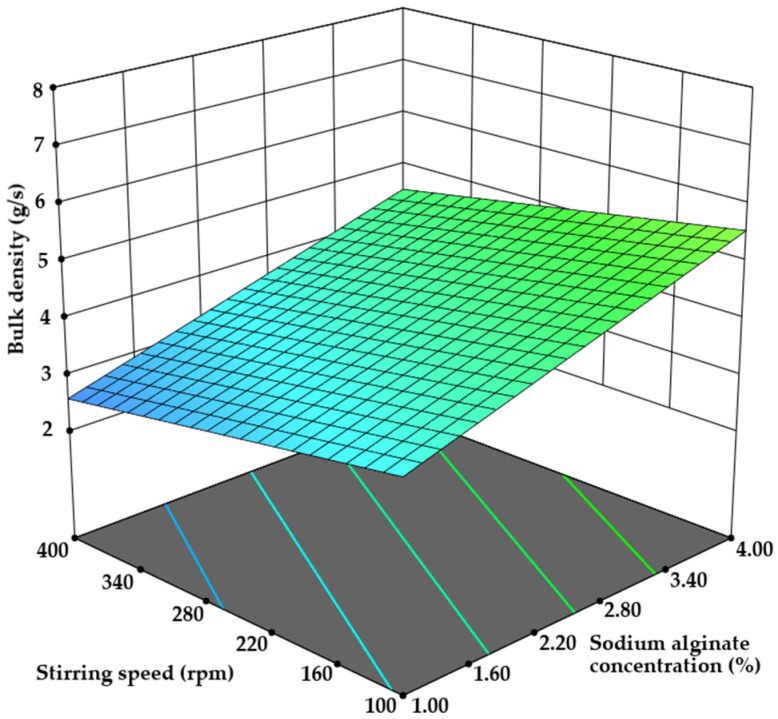
The influence of sodium alginate and stirring speed on the bulk density of microcapsules.

The assessment of microcapsule bulk density revealed values ranging from a minimum of 2.20 ± 0.10 g/s to a maximum of 8.40 ± 0.40 g/s. Spearman’s correlation analysis revealed a statistically significant correlation between sodium alginate concentration and microcapsule bulk density (r = 0.597), while other parameters showed much weaker correlations (*p* < 0.05). This result was further confirmed through careful data analysis by linear ANOVA. The model fit was determined to be significant, with a *p*-value of 0.019, and the only significant term was the concentration of sodium alginate (*p* = 0.0052). A four-fold increase in sodium alginate concentration used to form the emulsion resulted in a 63.43% higher bulk density of microcapsules (*p* < 0.05). However, during the study, several trends were observed: a five-fold slower flow rate decreased bulk density by up to 13.53% in some test samples, while a change in stirring speed from 100 to 400 rpm resulted in a nearly 22% decrease in some compositions. These results, though not statistically significant, provide valuable insights into the influence of process parameters on bulk density (Figure 8). Notably, a strong interrelationship exists among compression resistance, moisture content, and bulk density of microcapsules. The strongest correlation was observed between compression resistance and moisture content (r = 0.723), followed by bulk density and moisture content (r = 0.642), while the correlation between compression resistance and bulk density was moderate (r = 0.385, *p* < 0.05). Similar observations have been made by X. Sun et al., where reduced bulk density is attributed to lower moisture content. A dry solid tends to be looser when dissolved in water, leading to lower bulk density [48].

#### 3.2.4. Microcapsule Size Determination

**Figure 9:**
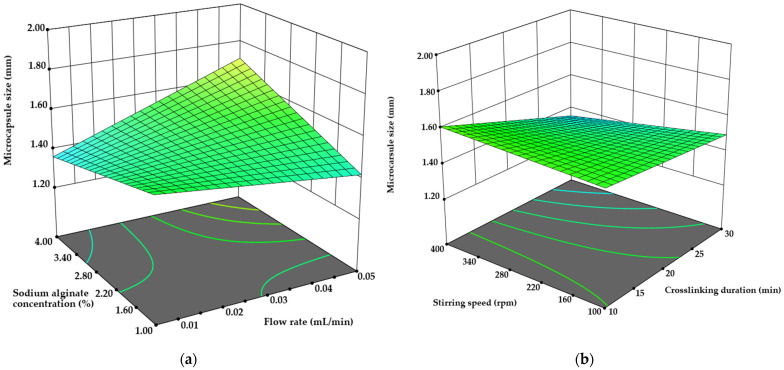
The influence of (**a**) sodium alginate concentration/extrusion flow rate and (**b**) crosslinking duration/stirring speed on the microcapsule size.

Microcapsule size had to be evaluated at this stage, as the size requirements for microcapsules depend on the administration route. For instance, in the case of parenteral injection, the particle size of microspheres typically ranges between 10 and 200 µm, whereas for oral administration, the size can be much broader [49,50]. The dimensions of the microcapsules were assessed through microscopic evaluation using Nis-Elements software, revealing a size range between 1.20 ± 0.06 mm and 1.91 ± 0.10 mm. Pearson’s coefficient showed a significant moderate correlation between stirring time and microcapsule size (r = 0.409), indicating that prolonged stirring may result in smaller microcapsule sizes. Similarly, the sequential sum of squares for the two-factor interaction ANOVA analysis (*p* = 0.045) demonstrated that stirring time, along with interactions between extrusion flow rate and sodium alginate concentration, significantly impacted microcapsule size (Figure 9).

There is no surprise regarding prolonged stirring durations with crosslinking agents and chitosan leading to smaller microcapsules and enhanced mechanical strength [29,51,52,53,54]. However, an increase in crosslinking duration from 30 to 120 min during the preparation of eucalyptus essential oil-containing sodium alginate microcapsules did not significantly impact microcapsule size, although a slight increase in particle size distribution by 7% was observed [54]. Additionally, when the microcapsule flow rate was shifted from 0.01 to 0.05 mL/min with a sodium alginate concentration of 1%, the resulting microcapsules were 7.74% larger. Conversely, at a 4% sodium alginate concentration, the microcapsules prepared with a flow rate of 0.01 mL/min were 20.4% smaller (*p* < 0.05 vs. 0.05 mL/min flow rate).

#### 3.2.5. Encapsulation Efficiency and Encapsulation Yield Determination

For encapsulation yield, a linear ANOVA technique was employed (*p* = 0.042), and the only significant term identified was the extrusion flow rate of the prepared emulsion. Increasing the flow rate from 0.01 mL/min to 0.05 mL/min resulted in a 4.89% higher encapsulation yield (*p* < 0.05). The overall encapsulation yield for microcapsules ranged from 84.12 ± 4.2% to 93.45 ± 4.5%. The optimal flow rate is composition-dependent [55]. High extrusion rates may prevent proper gelation of the alginate solution, leading to incomplete encapsulation and lower EY and EE. Conversely, slower extrusion flow rates generally yield higher EE and mechanical stability, but this can negatively affect the controlled release of encapsulated bioactive ingredients. It is worth noting that EY increased by 3.7% when the sodium alginate concentration was increased four-fold, though this finding was not statistically significant (*p* > 0.05).

**Figure 10:**
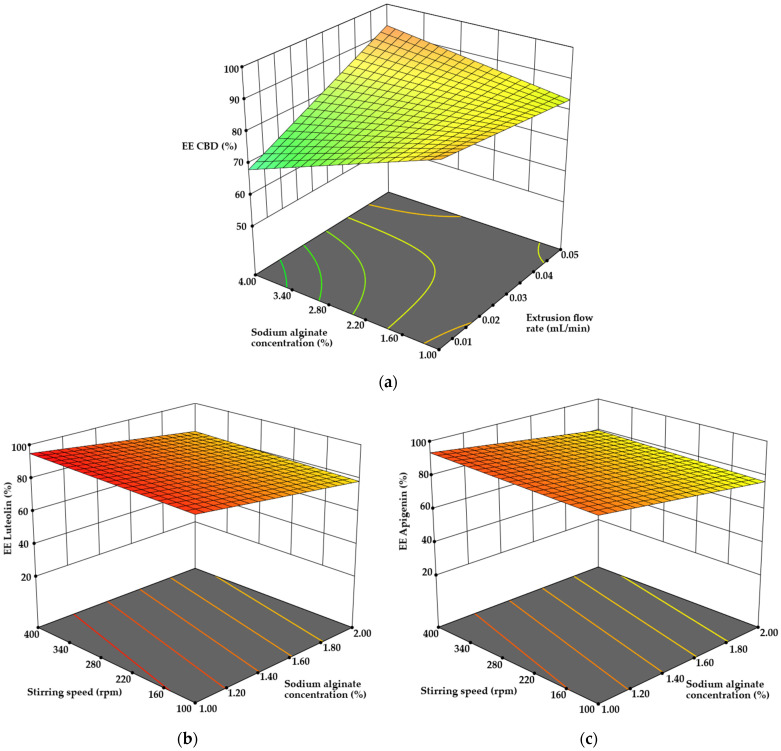
The influence of model terms on the encapsulation efficiency of (**a**) CBD, (**b**) luteolin, and (**c**) apigenin.

Encapsulation efficiency (EE%) for CBD, apigenin, and luteolin demonstrated some variability. The encapsulation efficiency of CBD was analyzed using sequential sum of squares for two-factor interactions ANOVA (*p* = 0.0476). The analysis revealed that only the interaction between sodium alginate concentration and extrusion flow rate had a significant impact on EE (*p* < 0.05), while other terms were insignificant (Figure 10a). Overall, the EE of CBD ranged from 55.23 ± 2.82% to 93.26 ± 4.71%. Increasing the sodium alginate concentration to 4% and raising the flow rate from 0.01 to 0.05 mL/min resulted in a 24.48% increase in EE. However, for a lower concentration of 1% sodium alginate, a similar change in flow rate only increased EE by 2.71%, indicating that higher sodium alginate concentrations enhance encapsulation capacity, likely due to improved emulsion viscosity. Nonetheless, higher extrusion flow rates may destabilize less viscous emulsions, resulting in the loss of encapsulated compounds during the extrusion process.

Similarly, the trends observed for apigenin and luteolin EE were consistent. Both datasets were analyzed using ANOVA for linear models (*p* < 0.05), and the only significant term was sodium alginate concentration. Doubling the concentration of sodium alginate resulted in a decrease in EE for both luteolin (13.45%) and apigenin (13.71%) (*p* < 0.05) (Figure 10b,c). Spearman’s coefficient indicated a strong relationship between sodium alginate concentration and encapsulation efficiency for both apigenin (r = 0.708) and luteolin (r = 0.707). Research by K. Essifi et al. also demonstrated that increasing the sodium alginate concentration from 3% to 6% led to a rise in encapsulation efficiency for gallic acid and crocin by 2.1–2.19%, though loading capacity decreased by 0.95–5.05%, respectively (*p* < 0.05). This suggests that the structural and physicochemical properties of the encapsulated compounds play a significant role in influencing EE and loading capacity [56].

#### 3.2.6. Cumulative Release In Vitro of Bioactive Ingredients Determination

Investigating the in vitro release of microcapsules in gastrointestinal media revealed cumulative release rates for apigenin, luteolin, and CBD across various samples. The release of active ingredients varied between the samples: CBD ranged from 22.2 ± 1.1% to 93.21 ± 4.7%, while apigenin exhibited a range of 20.52 ± 1.01% to 91.62 ± 4.61%, and luteolin ranged from 47.26 ± 2.41% to 93.63 ± 4.72%. All three datasets were analyzed using linear ANOVA with significance levels of 0.0397 and 0.0487 for CBD and apigenin release, respectively. The reduced release could be attributed to the denser crosslinked alginate network, which creates a more robust barrier, limiting the diffusion of CBD. This property may enhance the stability of the active compound during gastric transit, making it beneficial for targeted intestinal delivery, while apigenin release was significantly influenced by sodium alginate concentration only. However, no significant correlations were found between luteolin release and any microencapsulation process parameters, suggesting that other factors may have influenced the release of the compound.

An increase in stirring speed of 300 rpm resulted in a more than 30% decrease in cumulative release of CBD. Higher stirring speeds, along with the use of a crosslinking agent, were found to create smoother microcapsule surfaces with improved physicochemical characteristics of the shell, which subsequently affects the release of active materials [49]. Furthermore, a three-fold increase in sodium alginate concentration led to a 21.42% and 17.43% lower cumulative release of CBD and apigenin, respectively (*p* < 0.05). Notably, under the same conditions, the cumulative release of luteolin was 7.95% lower, though this result was deemed insignificant. These findings are consistent with other studies suggesting that increased sodium alginate concentration may negatively affect the release of bioactives [57,58]. However, there are studies where the effect of alginate polymer concentration on release was not significant, and release kinetics were largely dependent on the structure and physicochemical characteristics of the bioactive substance [56].

#### 3.2.7. Microcapsule Technological Optimization

These findings suggest that sodium alginate concentration likely influences the integrity of the microcapsules and the encapsulation efficiency of the respective compounds, whereas other factors such as stirring speed and duration appear to have a smaller effect. However, optimizing parameters such as stirring speed and flow rate could significantly influence the encapsulation yield and release kinetics of CBD, apigenin, and luteolin in microcapsules. Therefore, the optimization of the investigated parameters was carried out. Numeric optimization of these parameters was selected, and the level of importance of the outputs was determined. The cumulative release in vitro of active ingredients was of the highest importance (5+), followed by encapsulation efficiency (4+), encapsulation yield, moisture content, and bulk density, which were set to 3+, and microcapsule size to 2+.

The optimal technological conditions for microcapsules (further referred to as Composition B), based on the selected and prioritized outcomes, with a desirability value of 0.698, were determined as follows: sodium alginate concentration 1.8%, stirring speed 200 rpm, stirring duration 30 min, and flow rate 0.05 mL/min. Although the desirability value (0.698) is below the ideal threshold of 0.8, the optimized parameters demonstrated significant improvements in encapsulation efficiency and in vitro release profiles. Further refinement, such as incorporating stabilizers or modifying crosslinking conditions, could potentially enhance these outcomes. The predicted optimal values were: EE of CBD (76.61 ± 9.61%), apigenin (87.01 ± 3.01%), luteolin (89.04 ± 4.01%), EY (91.28 ± 3.71%), cumulative in vitro release of CBD (76.71 ± 19.17%), apigenin (70.10 ± 17.16%) and luteolin (71.61 ± 15.48%), moisture content (29.15 ± 5.10%), bulk density (4.11 ± 1.19 g/s), compression resistance (4122.92 ± 675.72 g), and microcapsule size (1.34 ± 0.144 mm).

The microcapsules were prepared following the predicted conditions, and the measurements were carried out. The obtained data did not differ significantly from the predicted data, so it was concluded that the optimal process characteristics for the extrusion of microcapsules were successfully determined.

### 3.3. Comparison of the Microcapsules after Composition and Process Optimization

#### 3.3.1. Release In Vitro Comparison

**Figure 11:**
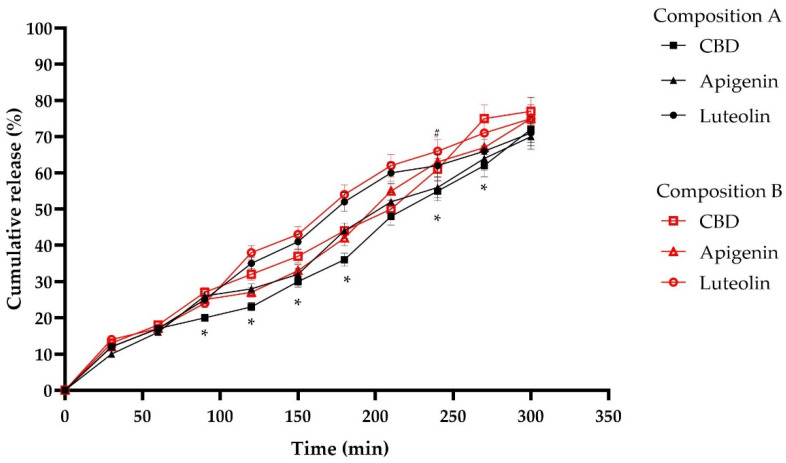
Cumulative CBD, apigenin, and luteolin release of microcapsules after composition optimization (Composition A, see Section 3.1.5) and technological process optimization (Composition B, see Section 3.2.7) in vitro. * *p* < 0.05 vs. CBD release of Composition A; ^#^ *p* < 0.05 vs. apigenin release of Composition A. n = 3–5.

The difference in cumulative release kinetics between the two compositions is shown in Figure 11. The results demonstrated that the optimization of technological processes (Composition B) improved the cumulative release of CBD. In a sample taken after 4.5 h, the difference between the release of the two different compositions was up to 13.3%. Unfortunately, no significant difference in luteolin release kinetics was observed. However, it was noted that after 4 h, the release of apigenin in vitro improved by 7.2 ± 2.1% (*p* < 0.05). This effect may have occurred due to the multiple emulsion used for the microcapsule extrusion. Since apigenin and luteolin were incorporated into the inner aqueous phase and dispersed into the lipophilic phase containing CBD, the release of saponins was affected the least.

The mathematical drug release models that best describe the release phenomena include the Higuchi model, zero-order model, first-order model, and Korsmeyer–Peppas model. Therefore, these models were applied to evaluate the kinetics and mechanisms of bioactive compound release from microcapsules. Luteolin and apigenin follow the zero-order release kinetics model (y = 0.2869x + 1.3214, R^2^ = 0.9818; y = 0.2119x + 3.2143, R^2^ = 0.9726), suggesting that the release rate is independent of the active compound concentration, while CBD follows a first-order kinetics model (y = −0.0013x + 1.9906, R^2^ = 0.992), indicating that the release is concentration-dependent [30].

#### 3.3.2. Microcapsule FT-IR Analysis

**Figure 12:**
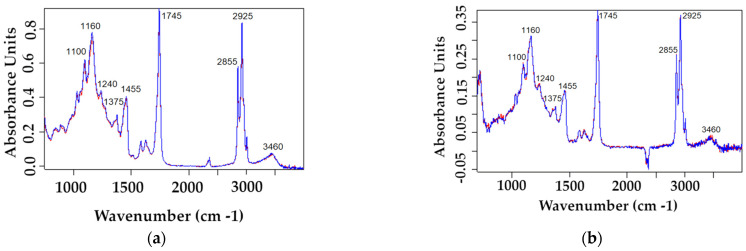
FT-IR spectrum of the chitosan–alginate microcapsules after (**a**) composition optimization and (**b**) technological process optimization.

Using the FT-IR spectrum we are able to analyze the peaks that represent the functional groups of the compounds in the chitosan–alginate microcapsules (Figure 12). In the spectrum the peaks 3460, 2925 and 2855 cm^−1^ show potential N–H stretching and C–H stretching, respectively, indicating that the -NH_2_ functional groups are present which are part of chitosan’s structure [27,59,60,61]. The C–H stretching at 2925 cm^−1^ indicates the vibration similar to aliphatic hydrocarbons, which are found both in alginate and chitosan structures, moreover this peak also corresponds to asymmetric stretching of -CH₂ (methylene groups) [27,59,60,61,62]. Specifically in chitosan the C–H stretching is present in the glucosamine units, while in alginate, they would arise from the mannuronic and guluronic acid units. The peak at 2855 cm^−1^ represents the possible symmetric stretching of -CH_2_ At 1745 cm^−1^ this peak indicates the presence of C=O stretching, mostly a carbonyl group that is present in aldehydes, ketones, esters, fats, oils and lipids, this peak could represent the carbonyl groups both in alginate structure, flavonoids (in this case flavones apigenin and luteolin), triterpenes, as well as in lipids within the Cannabis oil [27,60,61]. At 1455 cm^−1^ the peak shows a C–H stretching, specifically CH_2_ and CH_3_ bending (deformation) vibrations, it could also suggest asymmetric bending, which often found in lipids, fatty acids, and aliphatic chains, again present in Cannabis oil [27,60,61]. Another claim would be that this peak corresponds to CH₂ bending vibrations, which are common in chitosan and alginate structures [27,59,60,61,62]. A mild peak at 1375 cm^−1^ is visible which suggests that O–H bond is present, similar to a phenolic group that is found in flavones apigenin and luteolin and a peak following that at 1240 cm^−1^ might suggest a C–O stretching, which could correspond to the flavone skeleton or phenolic C–OH bond [61,63,64,65]. The peak at 1160 cm^−1^ represents the C–O stretching vibrations that are common in esters, this would explain the presence of the Cannabis oil and the fatty acids, and in the carboxylate groups or the glycosidic linkages in alginate [61]. Lastly, the peak at 1100 cm^−1^ corresponds to C–O–C stretching, suggesting the possible involvement of an oxane group, a cyclic ether, which is predominantly found in mentioned flavones, alginate and chitosan structures. Some of these peaks, notably at 2855, 1455, 1375 and 1240 cm^−1^ representing C–H, C=C, O–H, C–O–C bonds, respectively, might suggest the presence of triterpenes, phenols, and phenolic acids in microcapsules as well, judging by the height of the peaks [61,65].

#### 3.3.3. SEM Analysis

**Figure 13:**
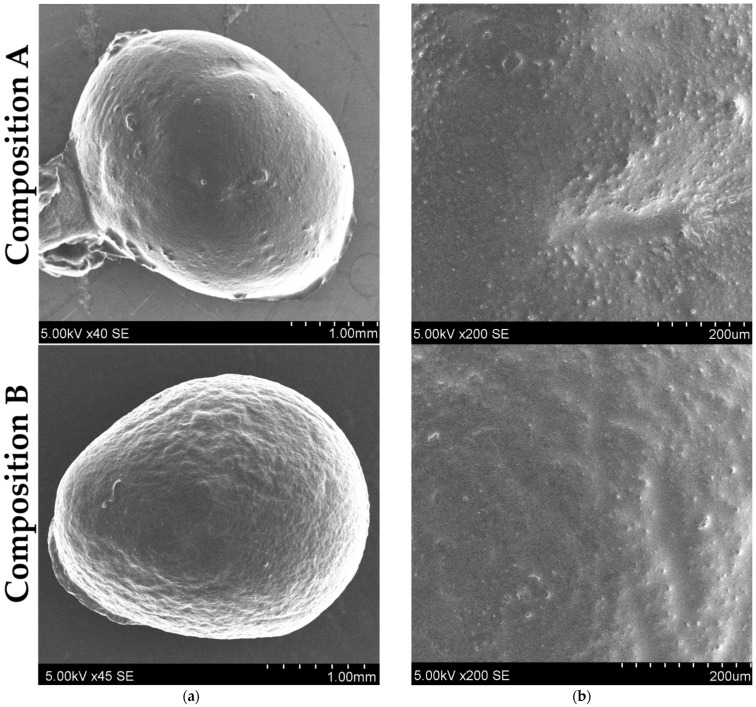
SEM images of the (**a**) shape and (**b**) surface of chitosan–alginate microcapsules after composition optimization (Composition A, see Section 3.1.5) and technological process optimization (Composition B, see Section 3.2.7).

The morphological analysis of the chitosan–alginate microcapsules, including their shape and surface, was evaluated by scanning electron microscopy (Figure 13). The microcapsules appeared to have a relatively spherical shape. As previously determined, stirring time and extrusion flow rate significantly affect both the microcapsule size and firmness. The surface of the microcapsules appeared smooth in some areas, while in others, it appeared rough and slightly wavy [60]. It has been reported that sodium alginate concentrations below 5% form microcapsules that shrink during drying due to fewer carboxyl groups being exposed for crosslinking. This can potentially lead to the collapse of the shell [66]. The dented surface and waviness are likely a result of the drying process of the microcapsules [27]. Additionally, the molecular weight of chitosan affects the morphology of the microcapsules: the higher the molecular weight, the rougher the surface of the microcapsules. This effect may be due to reduced diffusion ability into the microcapsule core [67].

The difference between Compositions A and B is attributed to the concentration of sodium alginate: microcapsules with 1.8% sodium alginate appeared firmer and smoother compared to those containing a 1.0% sodium alginate solution. Both compositions exhibited an approximately normal particle size distribution.

## 4. Conclusions

Chitosan–alginate microcapsules prepared using a W/O/W emulsion containing *A. annua* L. extract and CBD were successfully optimized through a two-step response surface methodology. The identified optimal composition and technological conditions were an inner aqueous phase of Tween 20 (0.1%), Span 80 (3.8%), CBD (3.8%), and *A. annua* L. extract (19.9%); an outer aqueous phase of Tween 20 (1.5%), 1.8% sodium alginate solution (48.5%), stirring speed of 200 rpm, stirring duration of 30 min, and a flow rate of 0.05 mL/min. The outcomes showed encapsulation efficiencies of CBD (80.32 ± 4.11%), apigenin (88.13 ± 3.13%), and luteolin (88.41 ± 4.17%), with cumulative in vitro releases of CBD (77.18 ± 4.40%), apigenin (75.12 ± 4.81%), and luteolin (75.32 ± 4.53%).

While most investigated factors significantly influenced these results, further research is needed to enhance the encapsulation efficiency and in vitro release of luteolin and apigenin. Incorporating excipients may also improve these properties. Nonetheless, the developed alginate–chitosan microcapsules containing *A. annua* L. plant extract with polyphenols, flavones, and cannabinoids provide a robust platform for the encapsulation of hydrophilic and lipophilic bioactives, potentially enabling their application in sustained drug release systems and functional food supplements designed for gastrointestinal health.

## Figures and Tables

**Table 1:** Surface response (optimal) design variables for the emulsion composition optimization.

Variable		Concentration Range (%)	
Code	Name	Minimum	Maximum
A	Tween 20 inner phase	0.00	0.60
B	Span 80	1.50	4.50
C	CBD	3.75	7.50
D	*Artemisia annua* L.	10.00	20.00
E	Tween 20 outer phase	0.50	1.50

**Table 2:** Surface response (optimal) design variables for the microcapsule preparation technique.

Variable		Unit	Range	
Code	Name		Minimum	Maximum
A	Stirring speed	rpm	100	400
B	Stirring duration	min	10	30
C	Flow rate	mL/min	0.01	0.05
D	Sodium alginate	%	1.0	4.0

**Table 3:** The terms equations of emulsion composition surface response D-optimal design.

Term	Aliased Terms Equations
Intercept	= Intercept − 0.0292 * AB + 0.0276 * AC − 0.069 * AD − 0.0256 * AE − 0.0172 * BC − 0.0287 * BD + 0.00847 * BE + 0.00078 * CD + 0.0137 * CE + 0.0216 * DE + 0.603 * A^2^ + 0.629 * B^2^ + 0.608 * C^2^ + 0.609 * D^2^ + 0.637 * E^2^
A	= A + 0.0253 * AB − 0.145 * AC + 0.0447 * AD + 0.0693 * AE − 0.0525 * BC − 0.0322 * BD − 0.0611 * BE − 0.0269 * CD − 0.0305 * CE + 0.127 * DE + 0.026 * A^2^ − 0.00414 * B^2^ − 0.0248 * C^2^ + 0.0296 * D^2^ + 0.043 * E^2^
B	= B − 0.0835 * AB − 0.0518 * AC − 0.0379 * AD − 0.0718 * AE − 0.146 * BC + 0.115 * BD − 0.00607 * BE + 0.027 * CD − 0.00578 * CE + 0.19 * DE + 0.00455 * A^2^ + 0.0521 * B^2^ + 0.00539 * C^2^ − 0.0315 * D^2^ + 0.0362 * E^2^
C	= C − 0.0517 * AB − 0.0891 * AC − 0.0285 * AD − 0.0341 * AE + 0.0322 * BC + 0.0283 * BD + 0.109 * CD + 0.0595 * CE + 0.0104 * DE − 0.0412 * A^2^ − 0.0365 * B^2^
D	= D − 0.0323 * AB − 0.0323 * AC − 0.048 * AD + 0.107 * AE + 0.0218 * BC − 0.00978 * BD + 0.181 * BE − 0.0982 * CD + 0.00737 * CE + 0.106 * DE − 0.0174 * A^2^+ 0.0542 * B^2^ + 0.0319 * C^2^ + 0.0943 * D^2^ + 0.0324 * E^2^
E	= E − 0.0731 * AB − 0.0262 * AC + 0.103 * AD − 0.0342 * AE − 0.00496 * BC + 0.181 * BD+ 0.0523 * BE + 0.00843 * CD − 0.0746 * CE + 0.0897 * DE + 0.0333 * A^2^ − 0.0403 * B^2^ + 0.0209 * C^2^ + 0.0577 * D^2^ + 0.0188 * E^2^

See A, B, C, D, and E references in Section 2.5, Table 1.

**Table 4:** The terms equations of microcapsule technology surface response D-optimal design.

Term	Aliased Terms Equations
Intercept	= Intercept + 0.0341 * AB − 0.0487 * AC − 0.0546 * AD − 0.0322 * BC − 0.0213 * BD + 0.0261 * CD + 0.605 * A^2^ + 0.587 * B^2^ + 0.602 * C^2^ + 0.594 * D^2^
A	= A + 0.0328 * AB − 0.0122 * AC − 0.00361 * AD − 0.0453 * BC − 0.0532 * BD + 0.0404 * CD + 0.016 * A^2^ + 0.0498 * B^2^ + 0.0157 * C^2^ + 0.0331 * D^2^
B	= B + 0.0207 * AB − 0.0358 * AC − 0.0429 * AD + 0.0252 * BC + 0.0273 * BD − 0.0714 * CD + 0.0496 * A^2^ + 0.0344 * B^2^ − 0.00649 * C^2^ − 0.0209 * D^2^
C	= C − 0.0268 * AB − 0.00762 * AC + 0.0252 * AD − 0.0115 * BC − 0.0716 * BD + 0.0312 * CD + 0.0401 * A^2^ + 0.0786 * B^2^ − 0.0977 * C^2^ + 0.0534 * D^2^
D	= D − 0.0392 * AB + 0.0326 * AC + 0.00931 * AD − 0.0726 * BC − 0.0301 * BD + 0.00106 * CD − 0.0171 * A^2^ + 0.0204 * B^2^ + 0.0246 * C^2^ − 0.0329 * D^2^

See A, B, C, and D references in Section 2.5, Table 2.

## Data Availability

The original contributions presented in this study are included in the article. Further inquiries can be directed to the corresponding author.

## Abbreviations

The following abbreviations are used in this manuscript:

W/O/W	Water-in-oil-in-water-type emulsion
SEM	Scanning electron microscopy
FT-IR	Fourier transform infrared spectroscopy
HPLC-DAD	High-performance liquid chromatography equipped with diode array detector
GC-FID	Gas chromatography with flame ionization detection
EE	Encapsulation efficiency
EY	Encapsulation yield
CI	Creaming index
CBD	Cannabidiol
